# Dataset of mobile learning effectiveness on learning Computer Programming in Community College

**DOI:** 10.1016/j.dib.2019.104525

**Published:** 2019-09-17

**Authors:** Hon-Sun Chiu

**Affiliations:** Hong Kong Community College, The Hong Kong Polytechnic University, Hong Kong

**Keywords:** Mobile learning, Computer Programming, Mobile apps, Academic performance, Mobile learning experience, Education, Pedagogical strategies, Community College

## Abstract

While learning through mobile devices, or mobile learning, has been proven feasible [1,2], its effectiveness is still in doubt as contradictory research results were observed [3–5]. In this dataset, the data collected from the experiments on mobile learning effectiveness is presented. The subject Computer Programming was used in the experiments because technical competence is one of the key success factors of mobile learning [6]. Computer Programming is an essential skill for all technical fields. It is therefore a compulsory foundation subject for all technical-related sub-degree programmes in Hong Kong Community College. Instead of comparing immediate pre-test and pro-test results, the entire subject performance of 1434 students in cohorts 2015 to 2017 was evaluated. By having different settings of mobile learning environment for each cohort, the effectiveness of mobile learning could be observed. The data collected was statistically analysed by one-way ANOVA with Turkey HSD post-hoc test. Students’ mobile learning experience was also evaluated by survey results using a 5-point Likert Scale questionnaire. The dataset in this paper should provide researchers and educators with further information on how mobile learning could be effectively implemented along with the pedagogical strategies.

**Table:** Specifications Table

**Subject**	Education
**Specific subject area**	Mobile learning
**Type of data**	Table
**How data were acquired**	Scores of subject assessments and survey using questionnaire
**Data format**	Raw, analysed, inferential statistical data
**Parameters for data collection**	The academic results of the control group and two experimental groups for comparison, and ratings of mobile learning experience.
**Description of data collection**	The academic results were obtained from the scores of all assessment components of students taking the subject. The ratings of mobile learning experience were collected by questionnaire feedback from students of cohort 2016.
**Data source location**	Hong Kong Community College, The Hong Kong Polytechnic University, Hong Kong
**Data accessibility**	Data is with the article

**Table:** 

**Value of the Data** • This dataset contains analysis on both academic performance and learning experience in mobile learning environment. It provides significant insights on how mobile learning could be effectively incorporated with pedagogical strategies in tertiary education. • Since contradictory conclusions were made by previous research works, researchers can obtain further information from this dataset regarding the effectiveness of mobile learning. In addition, educators and mobile app developers can better understand the effective use of mobile apps as an additional learning tool to enhance teaching quality. • The dataset in this article can be used to improve pedagogical practices in tertiary education. Further investigation may include implementation of mobile learning in different stages of learning process.

## 1 Data

Many research works have proven that mobile learning is feasible with various benefits such as diversifying the learning activities, supporting learning process, and synchronizing learning experience [1], [2]. However, the effectiveness of mobile learning on students' academic performance is still in doubt where contradictory research results were observed [3], [4], [5]. To evaluate the impact of mobile learning on students’ academic performance, the scores of all subject assessment components were analysed. The descriptive statistics and normality test on the scores are given in Table 1, Table 2, respectively. The assessment component “Overall (Before Exam)” represents the overall assessment result without considering the examination scores, while “Overall” represents the overall subject result. From Table 1, Table 2, the same observation is obtained for all cohorts of students. The scores of Test, Assignment 2 and Examination are normally distributed, where students had to submit these assessments in short time. For Assignment 1, Group Project and Participation which were take-home assessment, the distributions shift to right with higher mean scores. The score distribution of Overall (Before Exam) shifts to the right, and that of Overall subject result is normally distributed.

**Table 1:** Descriptive statistics on academic performance.

Assessment	Cohort	N	Mean	Std. Deviation	Std. Error	95% Confidence Interval for Mean		Min.	Max.
						Lower Bound	Upper Bound		
Test	2015	377	64.11	17.371	.895	62.35	65.87	19	100
	2016	383	63.27	16.933	.865	61.57	64.97	23	100
	2017	674	52.86	16.449	.634	51.62	54.11	16	99
	Total	1434	58.60	17.663	.466	57.68	59.51	16	100
Assignment 1	2015	377	87.79	9.582	.493	86.82	88.76	27	100
	2016	383	91.43	11.048	.565	90.32	92.54	7	100
	2017	674	86.54	11.363	.438	85.68	87.40	20	100
	Total	1434	88.18	11.018	.291	87.61	88.75	7	100
Assignment 2	2015	377	65.09	14.944	.770	63.58	66.61	25	100
	2016	383	64.25	16.683	.852	62.57	65.93	15	100
	2017	674	60.12	15.743	.606	58.93	61.31	10	100
	Total	1434	62.53	15.951	.421	61.70	63.36	10	100
Group Project	2015	377	80.76	10.837	.558	79.66	81.86	37	98
	2016	383	76.00	15.067	.770	74.48	77.51	14	100
	2017	674	78.16	12.467	.480	77.21	79.10	20	95
	Total	1434	78.26	12.936	.342	77.59	78.93	14	100
Participation	2015	377	87.71	16.955	.873	85.99	89.42	0	100
	2016	383	84.43	19.458	.994	82.47	86.38	0	100
	2017	674	85.96	19.658	.757	84.47	87.44	0	100
	Total	1434	86.01	18.954	.501	85.03	86.99	0	100
Overall (Before Exam)	2015	377	74.34	9.716	.500	73.36	75.33	44	96
	2016	383	72.98	10.906	.557	71.88	74.07	37	96
	2017	674	68.67	9.836	.379	67.93	69.41	38	95
	Total	1434	71.31	10.409	.275	70.77	71.85	37	96
Examination	2015	377	52.93	17.279	.890	51.18	54.68	10	99
	2016	383	54.98	18.798	.961	53.09	56.87	6	99
	2017	674	56.11	19.170	.738	54.66	57.56	5	100
	Total	1434	54.97	18.623	.492	54.01	55.94	5	100
Overall	2015	377	63.89	12.906	.665	62.58	65.19	33	98
	2016	383	64.25	14.106	.721	62.83	65.67	25	98
	2017	674	62.64	13.768	.530	61.59	63.68	28	98
	Total	1434	63.39	13.649	.360	62.69	64.10	25	98

**Table 2:** Normality test on assessment scores with Skewness and Kurtosis.

Assessment	Cohort 2015 (N = 377)		Cohort 2016 (N = 383)		Cohort 2017 (N = 674)	
	Skewness (Std. Error)	Kurtosis (Std. Error)	Skewness (Std. Error)	Kurtosis (Std. Error)	Skewness (Std. Error)	Kurtosis (Std. Error)
Test	−0.395 (0.126)	−0.566 (0.251)	−0.191 (0.125)	−0.651 (0.249)	0.134 (0.094)	−0.478 (0.188)
Assignment 1	−2.387 (0.126)	9.345 (0.251)	−4.566 (0.125)	28.585 (0.249)	−2.054 (0.094)	6.402 (0.188)
Assignment 2	−0.336 (0.126)	−0.273 (0.251)	−0.318 (0.125)	−0.140 (0.249)	−0.045 (0.094)	−0.295 (0.188)
Group Project	−1.339 (0.126)	3.389 (0.251)	−1.034 (0.125)	1.189 (0.249)	−1.353 (0.094)	1.962 (0.188)
Participation	−2.210 (0.126)	5.680 (0.251)	−1.754 (0.125)	3.192 (0.249)	−1.725 (0.094)	2.878 (0.188)
Overall (Before Exam)	−0.314 (0.126)	−0.248 (0.251)	−0.321 (0.125)	−0.194 (0.249)	−0.022 (0.094)	−0.186 (0.188)
Examination	−0.015 (0.126)	−0.548 (0.251)	−0.121 (0.125)	−0.626 (0.249)	0.087 (0.094)	−0.659 (0.188)
Overall	−0.086 (0.126)	−0.532 (0.251)	−0.142 (0.125)	−0.517 (0.249)	0.120 (0.094)	−0.570 (0.188)

The mean scores of the assessment components were statistically compared among the 3 cohorts using one-way ANOVA, with the result given in Table 3. There was no statistically significant difference among the 3 cohorts in terms of Participation (*p* = 0.058 > 0.05) and Overall subject result (*p* = 0.130 > 0.05). For other assessment components, the mean scores were further compared statistically using the Turkey HSD post-hoc test, where the result is given in Table 4. When comparing cohorts 2015 and 2016, no statistically significant difference was observed in the scores of Overall (Before Exam) (p = 0.150 > 0.05) and Examination (p = 0.282 > 0.05). When comparing with cohort 2017, the Overall (Before Exam) score of cohort 2017 is lower than the other cohorts where the difference is statistically significant (*p* = 0.000 < 0.05). The Examination score of cohort 2017 is higher than cohort 2015 with statistically significant difference (*p* = 0.022 < 0.05), but no statistically significant difference was observed when it is compared to cohort 2016 (*p* = 0.609 > 0.05).

**Table 3:** Statistical comparison on academic performance using one-way ANOVA.

Assessment	Sum of Squares	df	Mean Square	F	Sig.
Test	41993.340	2	20996.670	74.173	.000
Assignment 1	5914.017	2	2957.008	25.182	.000
Assignment 2	7529.018	2	3764.509	15.086	.000
Group Project	4326.678	2	2163.339	13.147	.000
Participation	2047.318	2	1023.659	2.857	.058
Overall (Before Exam)	9230.420	2	4615.210	45.226	.000
Examination	2445.260	2	1222.630	3.538	.029
Overall	758.855	2	379.427	2.040	.130

**Table 4:** Cohort analysis on academic performance using one-way ANOVA with Turkey HSD post-hoc test.

Dependent Variable	(I) Cohort	(J) Cohort	Mean Difference (I-J)	Std. Error	Sig.	95% Confidence Interval	
						Lower Bound	Upper Bound
Test	2015	2016	.848	1.221	.767	−2.02	3.71
		2017	11.252^a^	1.082	.000	8.71	13.79
	2016	2015	−.848	1.221	.767	−3.71	2.02
		2017	10.404^a^	1.077	.000	7.88	12.93
	2017	2015	−11.252^a^	1.082	.000	−13.79	−8.71
		2016	−10.404^a^	1.077	.000	−12.93	−7.88
Assignment 1	2015	2016	−3.640^a^	.786	.000	−5.48	−1.80
		2017	1.249	.697	.173	−.39	2.88
	2016	2015	3.640^a^	.786	.000	1.80	5.48
		2017	4.889^a^	.693	.000	3.26	6.52
	2017	2015	−1.249	.697	.173	−2.88	.39
		2016	−4.889^a^	.693	.000	−6.52	−3.26
Assignment 2	2015	2016	.842	1.146	.743	−1.85	3.53
		2017	4.974^a^	1.016	.000	2.59	7.36
	2016	2015	−.842	1.146	.743	−3.53	1.85
		2017	4.132^a^	1.011	.000	1.76	6.50
	2017	2015	−4.974^a^	1.016	.000	−7.36	−2.59
		2016	−4.132^a^	1.011	.000	−6.50	−1.76
Group Project	2015	2016	4.764^a^	.931	.000	2.58	6.95
		2017	2.605^a^	.825	.005	.67	4.54
	2016	2015	−4.764^a^	.931	.000	−6.95	−2.58
		2017	−2.158^a^	.821	.023	−4.08	−.23
	2017	2015	−2.605^a^	.825	.005	−4.54	−.67
		2016	2.158^a^	.821	.023	.23	4.08
Participation	2015	2016	3.280^a^	1.373	.045	.06	6.50
		2017	1.750	1.217	.322	−1.11	4.61
	2016	2015	−3.280^a^	1.373	.045	−6.50	−.06
		2017	−1.530	1.211	.416	−4.37	1.31
	2017	2015	−1.750	1.217	.322	−4.61	1.11
		2016	1.530	1.211	.416	−1.31	4.37
Overall (Before Exam)	2015	2016	1.366	.733	.150	-.35	3.09
		2017	5.673^a^	.650	.000	4.15	7.20
	2016	2015	−1.366	.733	.150	−3.09	.35
		2017	4.307^a^	.646	.000	2.79	5.82
	2017	2015	−5.673^a^	.650	.000	−7.20	−4.15
		2016	−4.307^a^	.646	.000	−5.82	−2.79
Examination	2015	2016	−2.051	1.349	.282	−5.22	1.11
		2017	−3.180^a^	1.196	.022	−5.99	−.38
	2016	2015	2.051	1.349	.282	−1.11	5.22
		2017	−1.130	1.190	.609	−3.92	1.66
	2017	2015	3.180^a^	1.196	.022	.38	5.99
		2016	1.130	1.190	.609	−1.66	3.92
Overall	2015	2016	−.362	.990	.929	−2.68	1.96
		2017	1.251	.877	.328	−.81	3.31
	2016	2015	.362	.990	.929	−1.96	2.68
		2017	1.613	.873	.154	−.43	3.66
	2017	2015	−1.251	.877	.328	−3.31	.81
		2016	−1.613	.873	.154	−3.66	.43

a The mean difference is significant at the 0.05 level.

To evaluate students’ mobile learning experience, the ratings collected from questionnaire survey were analysed and summarized in Table 5. The descriptive statistics and normality test of the ratings are given in Table 6. It is observed that the rating distributions of all items concentrate at high score corresponding to “Strongly Agree” and “Agree”. Most items have their mean ratings above 4, except item 5: “My classmates would suggest me to use mobile apps for learning” with mean rating 3.79, item 7: “I had experience in using mobile apps for learning in other subjects” with mean rating 3.41, item 15: “Using the app will stimulate my curiosity” with mean rating 3.98, and item 17: “Using the app will encourage discussion among classmates” with mean rating 3.87.

**Table 5:** Ratings of students’ mobile learning experience survey (N = 263).

No.	Question	Strongly Agree (5)	Agree (4)	Neutral (3)	Disagree (2)	Strongly Disagree (1)
1	I would find mobile apps useful in my learning.	124 (47.15%)	102 (38.78%)	31 (11.79%)	6 (2.28%)	0 (0.00%)
2	Using mobile apps enables me to accomplish learning activities more quickly.	109 (41.44%)	116 (44.11%)	31 (11.79%)	7 (2.66%)	0 (0.00%)
3	Using mobile apps increases my earning productivity.	102 (38.78%)	116 (44.11%)	39 (14.83%)	6 (2.28%)	0 (0.00%)
4	If I use mobile apps for learning, I will increase my chances of getting a better grade.	91 (34.60%)	100 (38.02%)	66 (25.10%)	6 (2.28%)	0 (0.00%)
5	My classmates would suggest me to use mobile apps for learning.	83 (31.56%)	75 (28.52%)	78 (29.66%)	22 (8.37%)	5 (1.90%)
6	My teachers would suggest me to use mobile apps for learning.	106 (40.30%)	105 (39.92%)	44 (16.73%)	7 (2.66%)	1 (0.38%)
7	I had experience in using mobile apps for learning in other subjects.	64 (24.33%)	74 (28.14%)	54 (20.53%)	47 (17.87%)	24 (9.13%)
8	I would prefer using mobile apps for learning in other subjects as well.	98 (37.26%)	111 (42.21%)	48 (18.25%)	4 (1.52%)	2 (0.76%)
9	The app is easy to use.	101 (38.40%)	104 (39.54%)	51 (19.39%)	6 (2.28%)	1 (0.38%)
10	It would be easy for me to pick up subject content by using the app.	95 (36.12%)	120 (45.63%)	42 (15.97%)	4 (1.52%)	2 (0.76%)
11	I can learn the subject content by using the app.	99 (37.64%)	122 (46.39%)	35 (13.31%)	7 (2.66%)	0 (0.00%)
12	I can evaluate my subject knowledge by using the app.	102 (38.78%)	122 (46.39%)	32 (12.17%)	6 (2.28%)	1 (0.38%)
13	I can find out my misunderstanding of subject content by using the app.	104 (39.54%)	107 (40.68%)	44 (16.73%)	7 (2.66%)	1 (0.38%)
14	Using the app will give enjoyment to me for my learning.	89 (33.84%)	97 (36.88%)	66 (25.10%)	9 (3.42%)	2 (0.76%)
15	Using the app will stimulate my curiosity.	82 (31.18%)	106 (40.30%)	65 (24.71%)	7 (2.66%)	3 (1.14%)
16	Using the app will lead to my exploration.	84 (31.94%)	121 (46.01%)	51 (19.39%)	6 (2.28%)	1 (0.38%)
17	Using the app will encourage discussion among classmates.	82 (31.18%)	89 (33.84%)	74 (28.14%)	13 (4.94%)	5 (1.90%)
18	The app is useful to my learning.	103 (39.16%)	117 (44.49%)	41 (15.59%)	1 (0.38%)	1 (0.38%)
19	I would recommend the app to my fellow classmates.	109 (41.44%)	97 (36.88%)	53 (20.15%)	4 (1.52%)	0 (0.00%)

**Table 6:** Descriptive statistics on students’ mobile learning experience survey (N = 263).

No.	Mean	Std. Deviation	Variance	Skewness (Std. Error)	Kurtosis (Std. Error)
1	4.31	.767	.588	−0.896 (0.15)	0.245 (0.299)
2	4.24	.763	.582	−0.807 (0.15)	0.283 (0.299)
3	4.19	.769	.592	−0.651 (0.15)	−0.121 (0.299)
4	4.05	.830	.688	−0.335 (0.15)	−0.89 (0.299)
5	3.79	1.039	1.080	−0.444 (0.15)	−0.557 (0.299)
6	4.17	.827	.684	−0.777 (0.15)	0.214 (0.299)
7	3.41	1.280	1.639	−0.357 (0.15)	−0.982 (0.299)
8	4.14	.817	.668	−0.764 (0.15)	0.566 (0.299)
9	4.13	.830	.688	−0.657 (0.15)	−0.053 (0.299)
10	4.15	.794	.631	−0.825 (0.15)	0.94 (0.299)
11	4.19	.763	.582	−0.7 (0.15)	0.124 (0.299)
12	4.21	.770	.594	−0.883 (0.15)	0.929 (0.299)
13	4.16	.824	.679	−0.766 (0.15)	0.225 (0.299)
14	4.00	.893	.798	−0.543 (0.15)	−0.215 (0.299)
15	3.98	.878	.770	−0.604 (0.15)	0.2 (0.299)
16	4.07	.798	.637	−0.578 (0.15)	0.129 (0.299)
17	3.87	.975	.950	−0.568 (0.15)	−0.105 (0.299)
18	4.22	.743	.552	−0.654 (0.15)	0.298 (0.299)
19	4.18	.804	.646	−0.521 (0.15)	−0.759 (0.299)

Item 20 of the questionnaire is an open-ended question. Most of the responses were related to the operations of the tailor-made mobile app. A few of them just consisted of the words “Good” and “Useful” that could be reflected from the ratings of other items. Therefore, the data related to item 20 is not included in this dataset.

## 2 Experimental design, materials, and methods

### 2.1 Context

As suggested by previous research, technical competence is one of the key success factors of mobile learning [6]. The subject Computer Programming was therefore selected for this study. Computer Programming is a compulsory foundation subject for all technical-related sub-degree programmes in Hong Kong Community College. The subject consists of six assessment components with different weightings contributing to the overall subject result, namely Test (16%), Assignment 1 (8%), Assignment 2 (8%), Group Project (13%), Participation (5%), and a Final Examination (50%). There were 1613 students taking this subject in cohorts 2015 to 2017. Among them, 1434 students attempted all assessment components and were the samples in this research.

For each cohort, students were distributed into different classes with similar class size of around 100 students per class. They were required to attend lecture and tutorial classes regularly throughout the semester. The same teaching schedule and same set of teaching materials were used. Apart from the test and final examination that were centrally arranged by the college, all other assessment components were released according to the same teaching schedule. Therefore, all students had the same amount of time to prepare and complete the assessments. Although the assessments were different in different semesters, they were designed to have the same level of difficulty.

### 2.2 Tailor-made mobile app

A mobile app called “CCN2042 C++” was designed for both iOS and Android devices. This mobile app was tailor-made to assist students' learning of the subject Computer Programming. It consists of illustration and explanation of basic programming techniques, simple programming exercises for students’ revision and practice, and small quizzes for checking their level of understanding.

Different from other similar mobile apps in the market, this tailor-made mobile app followed the teaching sequence of the subject throughout the semester. The content of the mobile app was also adopted from lecture notes and tutorial notes of the subject, so that students could reference easily. The quizzes of the mobile app were divided into more than 150 levels. Students had to complete a quiz correctly to unlock the quiz in the next level. Competition among students in reaching higher level could increase their motivation and engagement [7], [8].

### 2.3 Control and experimental groups

Cohort 2015 was the control group, where the tailor-made mobile app was not used. The mobile app was not even released to the market yet. Students were encouraged to have revision every week to consolidate their learning.

Cohort 2016 was the experimental group 1. The tailor-made mobile app was published via Apple Store and Google Play Store. Students were required to install the tailor-made mobile app and use it as an additional learning tool after each lecture. In every week, lecturers checked the progress of their students in using the mobile app, and reminded them to use the mobile app for revision according to the teaching schedule.

Cohort 2017 was the experimental group 2. There was no announcement of the tailor-made mobile app at the beginning of the teaching. Students were not forced to use it. There was no checking nor reminder related to the use of mobile app. Then at the end of teaching, students were advised to use the tailor-made mobile app for revision before the final examination.

### 2.4 Measurement

To evaluate the effect of mobile learning on students’ academic achievement, the scores of all assessment components in different cohorts were analysed. All descriptive statistics and inferential statistical analyses on the assessment scores were conducted in the software Statistical Package for Social Sciences (SPSS) version 25. The mean scores were statistically compared by one-way ANOVA test with a significance level of 0.05.

To evaluate students' mobile learning experience, a 5-point Likert Scale questionnaire with options “Strongly Agree”, “Agree”, “Neutral”, “Disagree” and “Strongly Disagree” was used. The questionnaire contained twenty items. Items 1 to 8 were designed to measure students’ perspective on mobile learning; items 9 to 19 were designed to measure the effectiveness on learning with the tailor-made mobile app; and item 20 was open-ended to collect other comments from students. Since only the students in cohort 2016 were forced to use the mobile app, the feedback was collected from students in cohort 2016 only. All students in cohort 2016 were invited to complete the questionnaire voluntarily and anonymously. They were well informed that the feedback from questionnaire would not affect their subject performance.
